# Evaluation of mobile phone addiction level and sleep quality in university students

**DOI:** 10.12669/pjms.294.3686

**Published:** 2013

**Authors:** Sevil Sahin, Kevser Ozdemir, Alaattin Unsal, Nazen Temiz

**Affiliations:** 1 Sevil Sahin, Sakarya University, School of Health Sciences, Sakarya, Turkey.; 2 Kevser Ozdemir, Sakarya University, School of Health Sciences, Sakarya, Turkey.; 3 Alaattin Unsal, Public Health Department, Osmangazi University, Eskisehir, Turkey.; 4 Nazen Temiz, Sakarya University, School of Health Sciences, Sakarya, Turkey.

**Keywords:** Mobile phone addiction, Sleep quality, University students

## Abstract

***Objective:*** To determine the mobile phone addiction level in university students, to examine several associated factors and to evaluate the relation between the addiction level and sleep quality.

***Methods:*** The study is a cross-sectional research conducted on the students of the Sakarya University between 01 November 2012 and 01 February 2013. The study group included 576 students. The Problematic Mobile Phone Use Scale was used for evaluating the mobile phone addiction level and the Pittsburgh Sleep Quality Index for assessing the sleep quality. Mann-Whitney U test, Kruskal-Wallis test and Spearman’s Correlation Analysis were used for analyzing the data.

***Results:*** The study group consisted of 296 (51.4%) females and 208 (48.6%) males. The mean age was 20.83 ± 1.90 years (min:17, max:28). The addiction level was determined to be higher in the second-year students, those with poor family income, those with type A personality, those whose age for first mobile phone is 13 and below and those whose duration of daily mobile phone use is above 5 hours (p < 0.05 for each). The sleep quality worsens with increasing mobile phone addiction level (p < 0.05).

***Conclusion:*** The sleep quality worsens with increasing addiction level. It was concluded that referring the students with suspected addiction to advanced healthcare facilities, performing occasional scans for early diagnosis and informing the students about controlled mobile phone use would be useful.

## INTRODUCTION

In line with the developing technology, the mobile phone use has become a part of daily life. Based on the data of the Turkish Statistical Institute, number of mobile phone subscribers in Turkey has increased by 5.8% in 2011 compared to previous year and become more than 65 million.^1^^,^^2^ It is known that the mobile phone use in Turkey is very common in well-educated young population.^1^

New generation mobile phones enable people not only to talk but also to connect to the virtual networks constantly from anywhere thanks to their computer and internet connection features. Currently, the mobile phones have become an important part of the daily life of the individuals and started to be considered as an imperative tool by the users.^3^^,^^4^ Constant mobile phone use has resulted in the concept of Nomophobia, in other words, the fear of being out of mobile phone contact. A study conducted in the UK in 2008 stated that 66% of the teenagers are troubled with the idea of losing their mobile phones.^4^

Griffiths (2003) suggested that everything that gives excitement causes addiction. From this perspective, the cause of addiction may be the fact that the mobile phone use is an exciting situation.^5^ Furthermore, according to the behavioral approach, if a behavior gives satisfaction or helps to get rid of a negative behavior such as tension or boredom then such behavior intensifies and the person keeps doing it for either taking pleasure or getting rid of a negative situation.^4^^,^^6^ In this case, mobile phone addiction both gives pleasure to the users as a result of the use and relieves them from stress or anxiety. Thus, it can be suggested that this situation causes mobile phone addiction.

 As a basic need of mankind, sleep is important for health and life quality at all ages. Sleep is defined as a basic element of physical growth and enhancement of the academic performance. Sleep quality is affected from several factors such as lifestyle, environmental factors, work, social life, economic situation, general health status and stress.^7^ One of the environmental factors that are believed to affect the sleep quality is the mobile phone use.

This study was intended to determine the mobile phone addiction level in the students of Sakarya University, examine several associated factors and evaluate the relation between the addiction and sleep quality.

## METHODS

The study is a cross-sectional research conducted on the students of the Sakarya University between 01 November 2012 and 01 February 2013. A total of 28,453 students are educated within 10 Faculties and 3 Institutes in the campus of the Sakarya University. In this study, the sample size was calculated as 524 students by using the sample size formula when the size of target population is known. After the departments which are School of Health, Faculty of Arts and Sciences, Faculty of Engineering, Faculty of Economics and Administrative Sciences were selected by casting lots, 576 students who were randomly selected from each class constituted the study group.

The survey form which was prepared by using the literature in line with the objective of the study^8^^,^^9^ includes some socio-demographic attributes of the students, several factors that are believed to be associated with the mobile phone use, and the questions on the Problematic Mobile Phone Use Scale and Pittsburgh Sleep Quality Index.

Before starting to collect data, required approvals were obtained from the University management and Faculty managers, and the students were gathered in the classes. The students were informed about the subject and objective of the study and verbal consents of the students were taken. Previously prepared survey forms were completed by the students under supervision. The rules stated in the Helsinki Declaration were complied in the stage of data collection.

The mobile phone addiction level was evaluated with the Problematic Mobile Phone Use Scale in our study. The scale was developed by Bianchi and Phillips.^10^ Validity and reliability study was conducted by Sar and Isiklar in Turkey.^4^ The scale consisted of 27 questions, 5 of which are Likert type. The scores to be obtained from the scale vary between 27 and 135. The higher scores denote to higher mobile phone addiction level.

In this study, the Pittsburgh Sleep Quality Index was used to evaluate the sleep quality. The index was developed by Buysse et al^11^ in 1989 and its validity and reliability study in Turkey was conducted by Agargun et al^12^ in 1996. Although the PSQI has 24 items, it is calculated as 19 items. The index has open-ended questions (e.g., During the last month, when did you go to bed?) and multiple-choice questions (e.g., During the last month, how was your sleep quality?), with answers such as very good, fairly good, fairly bad or very bad. Overall, 0–3 points are given for each question. The scores to be obtained in the scale vary between 0 and 21. The higher scores denote to poorer sleep quality.

Students who smoked at least one cigarette per day were defined as smokers, whereas nonsmokers were defined as men who had never smoked or who had not smoked in the past six months. In the current study, alcohol consumers were evaluated as those who had consumed at least one standard drink per week (one glass of raki / 1 cup vodka 1 cup gin / one glass of wine or one large glass of beer).

Those who defined themselves as uptight, enthusiastic, hasty, impatient in the study group were classified in “Type A personality” and those who defined themselves as quiet, calm, patient and organized were classified in “Type B personality”.^13^

The data obtained was evaluated in IBM SPSS (version 20.0) statistical package program. Mann-Whitney U test, Kruskal-Wallis test and Spearman’s Correlation Analysis were used for the analyses. The statistical significance level was accepted as p < 0.05.

## RESULTS

The study group consisted of 296 (51.4%) female and 208 (48.6%) male students. Their age varied from 17 to 28 with an average of 20.83 ± 1.90 years. 88 students (15.3%) are educated at the School of Health, 217 students (37.7%) at the Faculty of Arts and Sciences, 149 students (25.9%) at the Faculty of Engineering, and 122 students (21.2%) at the Faculty of Economics and Administrative Sciences. The scores obtained from the Problematic Mobile Phone Use Scale by the students varied between 27 and 135 with an average of 51.38 ± 15.30 points. The distribution of the average scores obtained from the Problematic Mobile Phone Use Scale by the students in the study group by specific characteristics is given in the Table-I.

**Table-I:** Distribution of the average scores obtained from the Problematic Mobile Phone Use Scale by the students in the study group by specific characteristics

*Socio-demographics*	*n*	*Score of Problematic Mobile Phone Use Scale* *Median (min-max)*	*Test value* *z/KW; p*	*Multiple comparison*	*p*
School					
School of Health	88	47.0 (27-94)	5.305; 0.151	-	-
Faculty of Arts and Sciences	217	49.0 (27-120)		-	-
Faculty of Engineering	149	45.0 (28-135)		-	-
Faculty of Economics and Administrative Sciences	122	49.0 (31-92)		-	-
Class					
1	167	48.0 (29-135)	9.538; 0.023	3-4	1.000
2	125	52.0 (27-130)		3-1	0.455
3	189	46.0 (27-108)		3-2	0.027
4	95	45.0 (30-89)		4-1	1.000
-	-	-		4-2	0.186
-	-	-		1-2	1.000
Sex					
Female	296	49.0 (28-120)	1.868; 0.062	-	-
Male	280	45.0 (27-135)		-	-
Age group					
≤19	141	50.0 (27-135)	4.932; 0.177	-	-
20	142	48.0 (29-130)		-	-
21	119	45.0 (27-88)		-	-
≥22	174	48.0 (28-108)		-	-
Family type					
Nuclear	464	47.5 (27-135)	2.145; 0.342	-	-
Extended	91	50.0 (30-94)		-	-
Divorced	21	48.0 (33-84)		-	-
Family income					
Poor 1	57	54.0 (32-94)	10.106; 0.006	2-3	0.247
Average 2	427	47.0 (27-135)		2-1	0.012
High 3	92	50.0 (27-130)		3-1	0.658
Where they stay					
With their family	75	46.0 (31-97)	3.076; 0.380	-	-
House	157	50.0 (27-135)			
Dormitory	316	47.0 (27-130)		-	-
Apart	28	48.5 (34-94)		-	-
Smoking					
Smoker	150	45.0 (27-130)	1.353; 0.176	-	-
Non-smoker	426	48.0 (27-135)		-	-
Alcohol consumption					
No	447	47.0 (27-135)	0.373; 0.710	-	-
Yes	129	49.0 (29-130)		-	-
Personality type					
A	310	51.0 (27-135)	4.975; 0.000	-	-
B	266	45.0 (27-108)		-	-
Total	576	48.0 (27-135)	-	-	-

The distribution of the average scores obtained from the Problematic Mobile Phone Use Scale by the students by the age for first mobile phone, mobile phone use frequency and duration of daily mobile phone use is given in the Table-II.

**Table-II:** Distribution of the average scores obtained from the Problematic Mobile Phone Use Scale by the students by some characteristics of mobile phone use

*Some characteristics of mobile phone use*	*n*	*Score of Problematic Mobile Phone Use Scale* *Median (min-max)*	*Test value* *z/KW; p*	*Multiple comparison*	*p*
Age for first mobile phone (year)					
≤13 (1)	163	49.0 (29-135)	8.515; 0.036	4-2	0.594
14 (2)	111	49.0 (27-97)		4-3	0.383
15 (3)	144	49.0 (27-96)		4-1	0.025
≥16 (4)	158	45.0 (27-94)		2-3	1.000
-	-	-		2-1	1.000
-	-	-		3-1	1.000
Frequency of mobile phone use (day)					
Once in a hour (1)	118	50.0 (27-94)	47.680; 0.000	4-3	0.257
A couple of times in a hour (2)	326	50.0 (27-135)		4-1	0.000
Once a day (3)	21	46.0 (30-80)		4-2	0.000
A couple of times in a day (4)	111	41.0 (27-94)		3-1	1.000
-	-	-		3-2	1.000
-	-	-		1-2	1.000
Duration of daily mobile phone use (hour)					
<1 (1)	151	45.0 (29-135)	34.153; 0.000	1-2	1.000
1-2 (2)	261	46.0 (27-94)		1-3	0.270
3-4 (3)	84	48.0 (27-120)		1-4	0.000
≥5 (4)	80	57.0 (34-130)		2-3	0.350
-	-	-		2-4	0.000
-	-	-		3-4	0.025
Total	576	48.0 (27-135)	-	-	-

As one person would state multiple reasons for using mobile phone, the reason of students for using mobile phone was inquired primarily in this study. The reasons for using mobile phone by the participants in the study group are given in the Table-III.

**Table-III:** The reasons for using mobile phone by the students

*Reasons for using mobile phone *	*n*	*%*
Calling family members	164	28.5
Calling friends	128	22.2
Texting	193	33.5
Using internet	66	11.5
Reading news	9	1.6
Playing game	8	1.4
Listening music	8	1.4
Total	576	100.0

Academic grade point average of the students was 2,55±0.56 (min: 1.00, max: 4.00) and no relation was found between the academic grade point average of the students and the scores obtained from the Problematic Mobile Phone Use scale (r_s_=-0.011; p=0.784).

**Fig.1:**
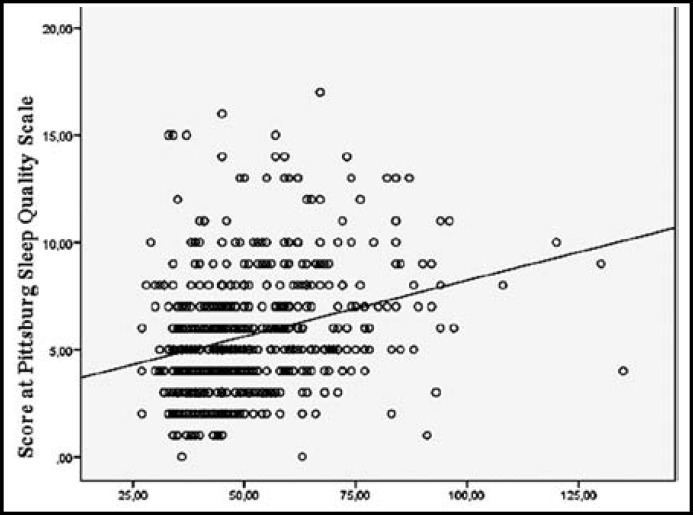
Distribution of the scores obtained from the Problematic Mobile Phone Use scale and the Pittsburg Sleep Quality Scale

The scores obtained from the Pittsburg Sleep Quality Scale by the participants in the study group varied between 0 and 17 with an average of 5.68 ± 2.86 points. A positive correlation was found between the scores obtained from the Problematic Mobile Phone Use scale and obtained from the Pittsburg Sleep Quality Scale by the students (r_s_=-0.297; p=0.000). The distribution of the scores obtained from the Problematic Mobile Phone Use scale and the Pittsburg Sleep Quality Scale is given in the Fig.1.

## DISCUSSION

The scores obtained from the Problematic Mobile Phone Use Scale by the students in our study vary between 27 and 135 with an average of 51.38 ± 15.30 points (median: 48.0). In the study of Zurkefly and Baharudin (2009), the average scale score was determined as 97.65 ± 36.65.^14^

No difference was found between the females and males with regard to the mobile phone addiction level (p>0.05). Similarly, Bianchi and Phillips (2005) stated that there is no difference between the females and males with regard to the mobile phone addiction.^10^ It shows that the female students use mobile phone for communication whereas the male students use mobile phone for different purposes such as watching a movie on the phone, playing games etc. in the same rates. Tasdemir (2012) determined that there is no difference between the sex and mobile phone use in a study on the mobile phone use by the university students.^15^ In the study of Beranuy et al. (2009) on problematic internet and mobile phone use by the university students, problematic internet and mobile phone use by the university students was found to be lower in the female students compared to the males.^9^

In the study group, the mobile phone addiction was determined to be higher in the students with poor family income (p < 0.05). Cankorkmaz (2011) reported that the levels of mobile phone use increase proportionally to the monthly pocket money of the students.^16^ Based on the TUBITAK’s study in 2006, analysis of mobile phone possession in households examined in terms of income demonstrated that there is at least one individual having a mobile phone in 27.4% of the households with low income while 98.1% of the individuals in high income group reported to have a mobile phone.^17^ The mobile phones which used to be preferred by only the high income society as they were extremely pricey have started to be used by most people as they get cheaper over the time.^18^

In this study, the mobile phone addiction level was higher in the students with type A personality (p < 0.05). In the study of Bianchi and Phillips (2005), extreme mobile phone use, spending time with mobile phone and the scores obtained from the Problematic Mobile Phone Use Scale were found to be higher in those with an outgoing personality.^10^

The mobile phone addiction level in the students whose age for first mobile phone was 13 and below was determined to be higher than the students whose age for first mobile phone was 16 and above (p < 0.05). In Uzgoren’s study (2012), the age for first mobile phone was 13-16 in the great majority of the students.^19^ Choliz (2012) reported that the mobile phone use was higher in the age group of 15-16 in the study conducted on the adolescents.^20^

In our study, when the frequency of mobile phone use during the day increases, the addiction level gets higher (p < 0.05). In the studies conducted on the university students, a large majority of the students believe that the mobile phones have a positive impact on daily life, consider them as an easy and fast communication method and think that being accessible at all times is a great convenience. The studies conducted also determined that the young people text at least 5 times in a day and want to reply all texts immediately. Based on these results, it can be suggested that increasing mobile phone use would cause more addiction.^8^^,^^18^

The addiction level of the students whose duration of daily mobile phone use is five hours and above is higher compared to other students (p < 0.05). Similarly, in the study of Zurkefly and Baharudin (2009), the students were reported to use mobile phone for five hours in a day on average.^14^ Choliz (2012) stated that mobile phone addiction was determined in the individuals whose duration of mobile phone use is approximately two hours.^20^

More than half of the students in the study group stated that their reason for using mobile phone is texting and calling family members. Aoki and Downes (2003) analyzed the mobile phone use in the university students and the impact of mobile phone use on the youth and revealed that the young people use mobile phone for many purposes such as feeling secure, financial interests, effective use of time and being in touch with their families and friends.^21^ Meurant’s (2007) study on mobile phone, electronic dictionary, text, computer and internet use of the freshman students in Korea reported that the university students use all researched information technology tools, primarily the mobile phone, commonly.^22^ Zurkefly and Baharudin (2009) stated in their study that 31.9% of the students said that they talk to their friends and 50.8% said that they talk to their family members.^14^

In this study, when mobile phone addiction level increases in the students, the sleep quality deteriorates (p < 0.05). In the study of Jenaro et al. (2007), the mobile phone use was associated with high anxiety and insomnia.^23^ In a study conducted in Saudi Arabia, extensive mobile phone use result in headache most frequently as well as sleep disorder, tension, fatigue and vertigo.^24^ Massimini and Peterson (2009) stated that a great majority of the students cannot sleep enough due to mobile phone use in at least one day of the week.^25^

***Limitations:*** The limitations of the study may include the facts that it is a cross-sectional study, it was conducted on the students of only one university and it is not possible to establish definitive diagnosis with used scales.

***Conclusion and suggestions: ***Mobile phone addiction is an important health problem in the university students. The sleep quality deteriorates with increasing addiction level. It was concluded that referring the students with suspected addiction to advanced healthcare facilities, performing occasional scans for early diagnosis and informing the students about controlled mobile phone use are required for the purposes of definitive diagnosis and treatment. It may be required to give priority to this matter, conduct more studies and evaluate them.

## Authors’ Contributions

**Sahin S, Ozdemir K, Unsal A: **Study concept and design.

**Sahin S, Ozdemir K, Temiz N: **Acquisition of subjects and data.

**Unsal A, Sahin S, Ozdemir K: **Analysis and interpretation of data:

**Sahin S, Ozdemir K, Unsal A, Temiz N: **Preparation of manuscript.

All authors have read and approved the final version of the manuscript.
